# Volunteering in a mental health service in Uganda: challenges and rewards

**DOI:** 10.1192/bji.2019.6

**Published:** 2019-11

**Authors:** Fiona Martin

**Affiliations:** ST4 General Adult Psychiatry, Northern Ireland Medical and Dental Training Agency, Psychiatry, Belfast BT8 7RL, UK. Email: fiona.martin2@northerntrust.hscni.net

**Keywords:** Transcultural psychiatry, low- and middle-income countries, human rights, education and training

## Abstract

Fiona Martin is a psychiatry trainee who spent 6 months volunteering in a mental health service in Uganda between her core and higher psychiatry training. In this article, she reflects upon this experience, including the challenges and rewards, and in particular the benefits to training of such an experience.

As a psychiatry trainee, I have always had an interest in global mental health. It has been my ambition to work in a mental health service in Sub-Saharan Africa. I was drawn to this area because I love the culture, and because there are many challenges to delivering mental healthcare, in particular, the scarcity of resources, stigma and maltreatment faced by many people. I decided to spend 6 months volunteering between my core and higher training, with my husband, who is a general practitioner. After initially struggling to find a suitable service, I arranged to volunteer in Kisiizi Hospital, Uganda, through Dr Maureen Wilkinson (Consultant Psychiatrist, Cheshire and Wirral Partnership NHS Foundation Trust), who has links with their well-established psychiatry department.

Uganda is a low-income country in East Africa with a rapidly growing population, currently approximately 42.8 million (The World Bank, 2017). The country has faced many challenges since independence, including significant instability. Poverty has had a significant adverse effect on health and service development (Molodynski *et al*, 2017).

Kisiizi, a rural village in the Rukungiri district, South West Uganda, sits in a beautiful green valley and is home to an amazing array of wildlife. The local waterfall provides hydro-electricity and a water supply. It is 380 km from the capital, Kampala, and 40 km from the district capital, Rukungiri; travel takes approximately 8 h and 1.5 h, respectively, on the potholed dirt roads (according to Google Maps). In this district, 86.2% of the population live rurally, the majority of whom are poor subsistence farmers (https://www.citypopulation.de/php/uganda-admin); 38% of the population live on less than $1.90/day (https://dataafrica.io/profile/rukungiri-uga). English is the official language in Uganda; however, there are over 40 local dialects, Rukiga being the one spoken in Kisiizi (World Atlas, 2017).

Uganda spends 9.8% of its gross domestic product on healthcare, of which less than 1% is spent on mental health. The majority of this is invested in the national psychiatric hospital, Butabika, in Kampala. It has 500 beds and is often overcrowded, and concerns have been raised about its conditions and practices (Molodynski *et al*, 2017). This is where all mental health professionals in the country are trained (Murray *et al*, 2015). There is patchy community mental health service cover (Molodynski *et al*, 2017).

Kisiizi Hospital is a private, not-for-profit, Church of Uganda hospital, partially funded by the Ugandan Government, the Church of Uganda and charitable donations. If patients cannot afford their healthcare, it is funded by the hospital's ‘Good Samaritan fund’: no patient should be denied care.

Working in a private service, serving a deprived population, prompted me to consider which investigations and treatments were really necessary. The hospital provides a not-for-profit health insurance scheme, through which local communities can group together to pay an annual fee. This covers emergency healthcare and other services, including admissions to the psychiatry department. The scheme currently has approximately 40 500 members.

In Kisiizi, and most other Ugandan hospitals, patients' friends and family, known as ‘attendants’, provide all their personal care and meals; for the occasional patients that do not have anyone to fulfil this role, nursing staff or, more commonly, other patient's attendants do so. I was struck by the attendants' dedication, and there were clear benefits to having friends and family so involved in patients' care.

The Christian faith is important to the Kisiizi hospital community. Every working day starts with chapel; as well as its religious purpose, this provides an opportunity for communication. My husband and I are not Christians, but we were welcomed as long as we respected their beliefs.

The hospital provided us with pleasant accommodation, alongside other volunteers from different cultural and occupational backgrounds, with whom we formed firm friendships, bonding over our shared experiences. We were also welcomed into the Kisiizi community and shared in lots of celebrations. We spent our time off exploring this fascinating and beautiful country.

The psychiatry department is aptly named The Ahumuza Centre, ‘Ahumuza’ meaning ‘place of comfort’ in Rukiga. The department was founded in 1997. It has a 28-bed in-patient unit and provides out-patient services to the local area as well as a liaison service to the main hospital. It provided care to almost 4000 patients last year. The current Ahumuza was opened in April 2017, funded by a UK-based charity, Jamie's Fund, replacing the old ward.

The team includes the experienced and dedicated Sister Nancy, a psychiatric clinical officer (PCO) (PCO's undergo 3 years of training in the assessment and management of psychiatric disorders, and can prescribe medication), a charge nurse and a small team of psychiatric and general nurses.

There is no doctor in the service, nor any clinical psychology or social work input. There is an occupational therapy assistant who provides activities for the in-patients. The PCO and psychiatric nurses have undergone basic training in psychological therapies and incorporate this into their care. There is a limited range of antipsychotic, antidepressant and anxiolytic medications available.

The current mental health legislation in Uganda is the Mental Health Treatment Act 1964, which is outdated and widely regarded as discriminatory (Kigozi *et al*, 2010); new legislation has been in the development stages for many years (Molodynski *et al*, 2017). However, in my experience, mental health legislation was not used, and, if necessary, patients were detained and treated under common law, depriving them of the safeguards that legislation provides.

There were differences in clinical presentations in Kisiizi compared with the UK. More than half of people with mental illness do not present to mental health services; therefore, those that do are not necessarily representative (Murray *et al*, 2015). Organic psychosis is more common, particularly HIV psychosis, with relatively higher rates of HIV (the adult prevalence in Uganda is 5.9%) (http://aidsinfo.unaids.org/). I saw more delirium occurring in young and otherwise healthy individuals due to increased rates of infectious diseases.

I also saw more patients with medically unexplained symptoms. This was probably owing to cultural differences in the expression of mental illness. The validity of the Western biomedical model of mental illness in low- and middle-income countries has been widely questioned. Patients from these countries tend to present more often with somatic symptoms (Patel *et al*, 2001; White, 2013).

I saw many patients with schizophrenia, schizoaffective and bipolar affective disorders, depressive and anxiety disorders, and alcohol misuse disorders, as in the UK. However, more patients had religious-themed psychotic symptoms, which I suspect was due to the greater prominence of religion in society.

Epilepsy is managed by mental health services in Uganda, as in much of Africa. Traditionally, the causes of both mental illness and epilepsy are regarded as bewitchment or spirit possession (Wilmshurst *et al*, 2014; Keikelame & Swartz, 2015).

I now realise that many of life's stresses are common across all cultures, with financial and relationship problems contributing to mental illness in Uganda, as in the UK. However, the contexts were often different: crop failures, famine, difficulty paying medical and school fees, land disputes and difficulties stemming from polygamous families.

In Uganda, the stigma around mental illness and epilepsy is enormous, in part because of the commonly held beliefs regarding causation by bewitchment or spirits. It is not uncommon that patients suffering from mental illness are hidden away from the world, shackled in squalid conditions and beaten. Patients and their relatives often experience significant shame, leading to reluctance to seek medical attention. Some people's lack of knowledge about mental illness means they are unaware that medical treatments exist (Molodynski *et al*, 2017).

Unfortunately, this stigma can also come from healthcare professionals, as well as being directed towards those that specialise in mental health. This can dissuade individuals from specialising, which contributes to staff shortages (Murray *et al*, 2015).

For many Ugandans, traditional or religious healers are the first port of call when they are unwell; it has been reported that 80% of patients in psychiatric units had consulted a traditional healer previously (Molodynski *et al*, 2017). There is little evidence for their treatments, some of which are potentially harmful. They can also delay urgent medical treatment. However, they have an excellent understanding of the psychosocial and cultural aspects of illness, and provide invaluable support to their patients. Some of their herbal medications appear to have powerful effects, but they have limited understanding of the pharmacology. Their training varies, but they hold an influential position within communities. I feel a collaborative working relationship with them is important (Ndetei, 2007; Mokgobi, 2014).

As there is not normally a doctor in the service, there was no pre-defined role for me. Technically, I was the most highly qualified member of staff, but I was very aware that I had the least experience and understanding of the service, and of mental illness in the Ugandan context. Initially, I spent time observing; then, when I felt ready, I worked alongside Sr Nancy, carrying out patient assessments, ward rounds, liaison assessments, and out-patient and outreach clinics. My relationship with Sr Nancy was based on mutual respect: there were times when I would seek advice from her, and *vice versa*.

In addition to clinical work, I gained invaluable experience in quality improvement, teaching and management. I identified some potential areas for improving clinical standards: with my colleagues, I carried out audits of prescribing, and then drafted guidelines. We also implemented new practices that I had experienced in the UK, including PRN prescription charts and a patient helpline. However, I was aware that it would not be appropriate to aim to simply replicate mental health services developed for high-income countries, but rather develop a culturally sensitive service.

I delivered teaching to a variety of audiences and participated in hospital management meetings. I had more responsibility than I had been accustomed to as a core trainee; however, I felt well supported. I was supervised remotely by Dr Wilkinson, who knows the service well, and on-site by Sr Nancy and consultants in other specialties. It was good preparation for my transition to higher training.

I experienced many challenges that exist in delivering a mental health service in rural Uganda. Some of these I had anticipated, such as lack of resources, stigma, maltreatment, and traditional and religious healers. While I was there, we had a patient admitted in shackles. I also saw a patient who had had holes drilled into their skull by a traditional healer for headaches, and had subsequently developed epilepsy.

When managing patients and relatives who believed mental illness was caused by bewitchment or spirits, I found the most effective approach was to listen to and respect their views, and then explain my own aetiological beliefs. We often agreed to disagree, but regardless I would encourage them to accept biomedical treatment. Many patients with these beliefs found they were compatible with accepting biomedical treatment.

My experience was that non-mental health specialist services, including primary healthcare services, had little appetite for managing mental illness, and therefore this service managed the majority of patients with mental illness, regardless of severity.

I faced several personal challenges. Initially, finding my role and gaining acceptance by my colleagues was difficult. I did not want to make anyone feel threatened, in particular Sr Nancy. These things were achieved through mutual respect.

There were times when I disagreed with my colleagues' practices, and I thought carefully about why I disagreed. An example was the over-sedation of patients. I worked together with my colleagues on this issue, as well as leading by example; with time, I was pleased to see an improvement. I feel one of my biggest achievements was leaving the department on good terms with my colleagues.

Working through an interpreter was challenging. I came to realise that there are limited words to express emotions in Rukiga; for example, there is no word for ‘sad’, which makes taking a psychiatric history difficult. It also led me to consider the effect this must have on people's experience of emotions. Cultural idioms of distress are ‘ways that cultural groups experience, understand and communicate suffering’; it is important to have an awareness of local idioms (Kohrt *et al*, 2016).

I realised the importance of having a good understanding of local culture. I relied on my Ugandan colleagues to advise me in this area. Many of the values that are important to me, such as respect, privacy and confidentiality, were not shared by my colleagues, which I found difficult. However, patients themselves did not seem to put much importance on privacy and confidentiality either. A possible explanation for this could be the collectivist culture in Uganda, which puts greater value on community involvement (Rarick *et al*, 2013).

Overall, I am extremely impressed at how this service manages despite the challenges it faces. The service has overseen the formation of patient groups, with patients providing invaluable support to each other in the community. I feel locally developed psychosocial interventions such as this are important. While I was there, I saw lots of patient success stories.

In going to Uganda, I was under no illusions that I was going to make any earth-shattering changes to their mental health service. However, I am hopeful that together with my colleagues we were able to make modest improvements.

But one thing I am certain of is the significant positive influence that the experience has had on me, both personally and professionally. First, it was an amazing experience to live and work in another culture: we were warmly welcomed into the Kisiizi community. I learned a lot from Ugandan culture, with the genuine friendliness and community spirit. Their culture of supporting each other is in sad contrast to our increasingly isolative lives in the UK.

I also gained invaluable experience in clinical and communication skills, teaching, quality improvement and management. My team working skills were tested like they never had been before, and I was faced with many ethical dilemmas. I developed a greater understanding of the cultural aspects of psychiatry. I have taken my newfound knowledge and skills back to my work in the UK, as I complete my higher training in general adult psychiatry. I was inspired by some of the mental health professionals I met in Uganda, who had developed innovative ways to deliver good-quality mental healthcare to the communities they serve, despite the barriers. I would highly recommend the experience of working in a mental health service in another culture to any other mental healthcare professionals considering it.
